# A comparison of the dose distributions from three proton treatment planning systems in the planning of meningioma patients with single‐field uniform dose pencil beam scanning

**DOI:** 10.1120/jacmp.v16i1.4996

**Published:** 2015-01-08

**Authors:** Paul J. Doolan, Jailan Alshaikhi, Ivan Rosenberg, Christopher G. Ainsley, Adam Gibson, Derek D'Souza, El Hassane Bentefour, Gary Royle

**Affiliations:** ^1^ Department of Medical Physics and Bioengineering University College London London UK; ^2^ Department of Radiotherapy University College London Hospital London UK; ^3^ Department of Radiation Oncology University of Pennsylvania Philadelphia PA USA; ^4^ Ion Beam Applications Louvain la Neuve Belgium

**Keywords:** proton therapy, particle therapy, treatment planning, planning comparison

## Abstract

With the number of new proton centers increasing rapidly, there is a need for an assessment of the available proton treatment planning systems (TPSs). This study compares the dose distributions of complex meningioma plans produced by three proton TPSs: Eclipse, Pinnacle^3^, and XiO. All three systems were commissioned with the same beam data and, as best as possible, matched configuration settings. Proton treatment plans for ten patients were produced on each system with a pencil beam scanning, single‐field uniform dose approach, using a fixed horizontal beamline. All 30 plans were subjected to identical dose constraints, both for the target coverage and organ at risk (OAR) sparing, with a consistent order of priority. Beam geometry, lateral field margins, and lateral spot resolutions were made consistent across all systems. Few statistically significant differences were found between the target coverage and OAR sparing of each system, with all optimizers managing to produce plans within clinical tolerances (D2<107% of prescribed dose, D5<105%, D95>95%, D99>90%, and OAR maximum doses) despite strict constraints and overlapping structures.

PACS number: 87.55.D‐

PACS number: 87.55.D-

### I. INTRODUCTION

The physical properties of protons can, in some cases, allow for an improved dose distribution compared with conventional X-ray radiotherapy.[(1–3)] The proton Bragg peak, characterized by a sharp distal penumbra, makes it possible to limit the dose to critical structures in ways that are not possible with X-ray radiotherapy. This distinct advantage, coupled with falling costs, has led to a significant increase in the interest in, and availability of, proton beam therapy. Between 2010 and 2013, nine new centers opened worldwide, with a further 27 planned to open by the end of 2016.[(4)] The primary mode of treatment for these new centers will be pencil beam scanning (PBS) rather than the traditional passive scattering techniques, driven by improved efficiency (passive techniques require patient-specific apertures and compensators for each field) and potentially lower neutron dose[(5)] (because of the absence of physical beam spreading

[a] Corresponding author: Paul J. Doolan, Department of Medical Physics and Bioengineering, University College London, Gower Street, London, WC1E 6BT, UK; phone: +44 (0)20 7679 0262 ; fax: +44 (0)20 7679 0255; email: paul.doolan.09@ucl.ac.uk and shaping devices in the nozzle). To date, a single vendor has constructed more than half of all proton therapy clinical facilities worldwide, with five already treating daily with PBS.[6] The majority of new contracts signed are for centers treating fully PBS, with only a few new centers purchasing double scattering for the treatment of mobile tumors.

PBS treatments use two common planning techniques: (i) single field uniform dose (SFUD), where each field is individually optimized to deliver a uniform dose distribution to the target; and (ii) intensity-modulated proton therapy (IMPT), where the final dose distribution is the result of contributions from multiple fields, each of whose individual contribution to the target dose distribution is nonuniform.[7] Both of these approaches have a large number of degrees of freedom, with the planner being able to control the position, energy, and intensity of every pencil beam. As such, an inverse optimization planning process is required.

Inverse optimization involves the minimization of a function that quantifies aspects of the dose distribution and specifies trade-offs between target volume coverage and organ at risk (OAR) sparing.[8] The routine to minimize this cost function is similar between all systems and thus unlikely to cause differences between plans in and of itself. However, each software has a different method of preweighting and positioning spots prior to the minimization step, so it is expected there will be differences between the plans produced by each system.

There have been many publications of treatment planning comparisons between photons and protons (for a selection:[9–13]; specifically for meningiomas:[14,15]) and for different proton therapy techniques.[16–19] A number of publications exist on the theory and accuracy of the dose calculation algorithms for two of the systems analyzed in this work (Eclipse[20–24] and XiO[25,26]). For Pinnacle$^3$, only the theoretical basis is available[25,27–30] because, at the time of the study, Philips had yet to receive regulatory clearance for the software. While there have been numerous comparisons of commercial photon treatment planning systems (TPS),[31–35] we are not aware of any publications comparing the plans produced by different proton TPSs. This is largely because proton TPSs were typically built in-house, but the rapid expansion of proton therapy has led the major photon TPS vendors to develop or purchase their own modules. This study compares the plans produced by three proton TPSs, Eclipse (Varian), Pinnacle$^3$ (Philips) and XiO (Elekta), for a set of ten meningioma patients in which as many variables as possible were kept consistent. It is the hoped that such an assessment will provide additional information to the procurement process for new proton centers and will encourage further proton TPS development.

Meningiomas account for 13%–26% of all primary intracranial tumors.[36] It has been shown that proton therapy can decrease the dose to OARs in meningioma patients.[37] This advantage of proton therapy, together with the difficulty of treating a volume that has a number of overlapping or surrounding critical structures, make these suitable cases on which to conduct a dose distribution comparison of different systems. Fixed horizontal SFUD fields were employed in the study because, at the time of writing, they are probably the most common implementation of spot scanning proton beams due to their relative robustness over IMPT fields to range uncertainties.[38,39] Although we allowed couch rotations, by removing the additional degrees of freedom provided by IMPT plans, differences between systems could be more easily identifiable.

A full comparison of the entire TPS of each system would be impractical, due to the size, complexity, and number of so many different features on each system. Rather than attempt such a task, that would quickly become out of date as the TPS evolves, our preliminary work focused on a very specific case (SFUD for meningioma patients). Even then, there were restrictions to the approach we could take. Firstly, the vendors have proprietary rights to their software and so it was not possible to know the internal details of the algorithms. Second, the complexity of the systems makes it very difficult to single out a specific component for testing. As such, we took a pragmatic approach of comparing the best plan that each system could produce with the same clinical constraints. Such an approach has both advantages (e.g., it allows the use of options offered by each TPS) and disadvantages (e.g., there are different implementations between TPSs and planners). The accuracy of each dose calculation algorithm is an important topic, but it is something that we decided not to investigate in this work due to the scale and depth required by the task. Also, phantom studies are not purely a reflection of the inherent accuracies of the dose calculation algorithms themselves, but also of how well the respective beam models have been configured; the latter can be improved with experience. Given our relative inexperience with each system, we decided to avoid this ambiguity and simply assessed the beam models by their abilities to reproduce the measured input data; all TPSs were found to be capable of doing this within clinical tolerances.[40]

The comparison was formed of two main analyses of 10 patient plans for each system: (i) the DVH metrics for the target and OARs; and (ii) an assessment of the uniformity and distribution of spot weights. No qualitative features (e.g., ease of use, visualization features) were compared to avoid the possibility of subjectivity. Although there are numerous ways to compare different TPSs, we believe that this is the first study, albeit a preliminary one, to explicitly compare clinical implementations of different TPSs for proton therapy.

### II. MATERIALS AND METHODS

#### A. Treatment planning systems

This intercomparison study was conducted at a dose planning level. SFUD plans, using an horizontal fixed beam geometry, were produced for three TPSs available to us:

i) Pinnacle$^3$, Philips Radiation Oncology Systems (Andover, MA), version 9.1.00. This was a nonclinical research system, as Philips had not yet received regulatory clearance for this software;
ii) Eclipse, Varian Medical Systems (Palo Alto, CA), version 10.0.39;
iii) XiO, Elekta CMS (St. Louis, MO), version 4.70.00.

#### B. Beam commissioning

A fair comparison requires all systems to have the same beam data. Therefore, raw PBS beam data from the Hospital of the University of Pennsylvania's horizontal fixed beamline, an Ion Beam Applications machine, were added to each system and commissioned according to the requirements and tools of each TPS. The beam models were tuned within each system until the differences from the input data were within clinical tolerances.[40] A consistent minimum spot monitor unit (MU) of 0.021 MU was enforced on all systems to ensure the plans were deliverable (so that the statistical error on the spot dose, as dictated by the charge measurement resolution of the monitor chambers in the nozzle, is within 1%). These MU constraints are dealt with during postprocessing, with all systems rounding equivalently (the impact of which is discussed in depth elsewhere[41]). The Hounsfield unit (HU) to stopping power calibration curve was determined using a stoichiometric calibration[42] and was identical between all three systems. As one patient had titanium clips inside the target, the HU had to be overridden in contouring because of saturation in the image.

### C. Patients

Ten meningioma patients, treated at our institution using a RapidArc (Varian Medical Systems) X-ray radiotherapy treatment, were selected for the comparison. The case load included tumors with and without overlapping structures, of different sizes (target structures ranged from 45.5 to 248.4 $cm^3$) and grades. Full details can be found in Table 1.

Table 1. Patient and tumor characteristics.

| *Patient* | *Age / Years* | *Grade*[a] | *Details* | *Beam Direction(s)* |
|---|---|---|---|---|
| 1 | 57 | 2 | Parafalcine | LSO, RSO |
| 2 | 67 | 2 | Frontal | Vertex |
| 3 | 40 | - | Cavernous sinus | LLat, LSO |
| 4 | 67 | 1 | Cavernous sinus | LLat, LSO |
| 5 | 40 | 1 | Sphenoid wing | RLat, RSO |
| 6 | 31 | 2 | Cavernous sinus | LLat, LSO |
| 7 | 19 | 2 | Sphenoid wing (brain invasion) | LLat, LSO |
| 8 | 40 | - | Cerebellopontine angle | RLat, RSO |
| 9 | 55 | - | Left orbital apex/sphenoid | LLat, LSO |
| 10 | 62 | - | Cavernous sinus | LLat, LSO |

[a] Grade only measured for patients in which a biopsy was practically possible.
L = left; R = right; S = superior; O = oblique; Lat = lateral.

### D. Target volumes, OARs, and constraints

The gross tumor volume (GTV) and clinical target volume (CTV) were outlined according to departmental protocol. Each CTV was expanded by 5 mm to a pencil beam scanning planning target volume (PBSTV). This expansion, which is 2 mm larger than that employed to construct the departmental planning target volume used for X-ray radiotherapy planning, is to make the plans sufficiently robust with respect to range uncertainties. Conventionally, margins associated with range uncertainties should only be applied in the proton field direction, but none of the systems allowed for the production of beam-specific target volumes. Additionally, it was much more convenient for the planner to work with a single target volume. The use of a consistent margin was appropriate for all patients as the distances to the target distal edge were approximately similar (9.9–13.9 cm) and thus the range uncertainties (a prescription of 3.5% $\times$ range + 1 mm was used at our institution) did not vary much (4.5–5.9 mm).

The dose prescription was 50.4 Gy in 28 fractions. The dose-volume constraints for the coverage of the PBSTV, together with the OAR constraints, are detailed in Table 2. All OAR constraints apply to the planning risk volumes (PRVs), formed by 3 mm expansions from the corresponding structures, to account for patient motion. Although no specific constraints were applied, attempts were made to keep the brain dose as low as possible without sacrificing target coverage and the mean dose to the healthy brain was assessed.

Table 2. Dose-volume constraints and coverage statistics. All OARs refer to PRVs. The bracketed values in the constraint column are the percentages of the prescribed dose (50.4 Gy). The bracketed values in the Eclipse, Pinnacle$^3$, and XiO columns are the number of patients who failed to meet the criteria.

| *Structure* | *Metric* | *Constraint / Gy* | *Eclipse / Gy* | *Pinnacle$^3$ / Gy* | *XiO / Gy* | *P* |
|---|---|---|---|---|---|---|
| PBSTV | D2 | < 53.9 (107%) | 52.4±0.1 (0) | 51.4±0.3 (0) | 51.0±0.1 (0) | $1.7\times10^{-4}$[a] |
| | D5 | < 52.9 (105%) | 52.0±0.1 (0) | 50.9±0.2 (0) | 50.7±0.1 (0) | $5.6\times10^{-7}$[a] |
| | D95 | > 47.9 (95%) | 46.5±0.8 (7) | 48.0±0.6 (2) | 48.0±0.6 (1) | 0.21 |
| | D99 | > 45.4 (90%) | 43.2±1.6 (6) | 43.1±1.9 (3) | 43.7±2.1 (4) | 0.97 |
| | V95 | - | 91.3±1.9% | 95.8±1.4% | 95.6±1.0% | 0.041 |
| BStem | Max | < 55 | 41.5±6.8 (0) | 41.7±6.5 (0) | 41.0±6.5 (0) | 1.0 |
| | Mean | | 20.0±4.0 | 14.7±3.1 | 21.2±4.0 | 0.43 |
| R Glo | Max | < 45 | 22.0±4.5 (0) | 14.9±4.8 (0) | 18.6±4.9 (0) | 0.58 |
| | Mean | | 6.1±2.7 | 3.6±2.5 | 4.7±3.0 | 0.81 |
| L Glo | Max | < 45 | 33.8±4.8 (1) | 31.7±5.5 (1) | 31.6±4.9 (0) | 0.94 |
| | Mean | | 12.4±2.9 | 8.8±2.8 | 7.0±1.7 | 0.31 |
| R ON | Max | < 50 | 31.8±5.7 (1) | 27.4±6.0 (0) | 33.1±5.3 (1) | 0.76 |
| | Mean | | 15.5±3.8 | 10.3±3.3 | 13.9±3.6 | 0.58 |
| L ON | Max | < 50 | 42.8±4.9 (2) | 41.9±5.2 (2) | 43.5±4.6 (3) | 0.98 |
| | Mean | | 34.3±5.4 | 32.8±5.8 | 32.0±5.1 | 0.95 |
| O Chi | Max | < 50 | 44.2±4.9 (0) | 44.6±4.9 (1) | 45.9±4.1 (2) | 0.97 |
| | Mean | | 38.1±5.5 | 37.3±5.6 | 39.7±4.9 | 0.95 |
| R Lens | Max | < 6 | 6.7±2.3 (5) | 1.7±1.0 (1) | 1.2±0.4 (1) | 0.024[a] |
| | Mean | | 2.7±1.4 | 0.5±0.3 | 0.3±0.1 | 0.094 |
| L Lens | Max | < 6 | 11.9±3.3 (6) | 6.2±3.2 (4) | 2.6±0.6 (5) | 0.063 |
| | Mean | | 6.6±2.2 | 3.0±2.1 | 1.0±0.3 | 0.092 |
| Brain | Mean | | 8.2±0.6 | 6.5±0.6 | 8.9±0.6 | 0.022[a] |

[a] Significant.

PBSTV = pencil beam scanning target volume; BStem = brainstem; R/L Glo = right/left globe; R/L ON = right/left optic nerve; O Chi = optic chiasm; R/L Lens = right/left lens; Brain = healthy brain.

### E. Planning strategy

The study objective was to compare clinically equivalent plans between TPSs, individually optimized for each system. The decision was therefore made to try to give the planners the freedom they would have were they to use each TPS for real planning (i.e., allow choice of objectives and priorities), but with some artificial constraints imposed to keep the test fair. Similar approaches have been used by other authors conducting TPS comparison studies.[31,33]

Beam geometries, patient isocenters, and outlined structures were made consistent between all systems. The beam arrangements used are detailed in Table 1. The same clinical constraints (Table 2) were used for all patients; however, the choice of numerical objectives that achieves these goals differs between systems and so was not fixed. Symmetric lateral spot spacings were defined according to the spot size (the larger of the X and Y directions was selected) at the most distal layer of the target, such that there is sufficient overlap between neighbors to avoid appreciable dose ripples. Lateral margins were then set to be equal to this spacing to ensure at least one additional ring of spots was located outside the PBSTV, reducing the possibility of highly weighted spots close to the target edge.

It was not possible to define the layer spacing in an identical fashion in each system, although Pinnacle$^3$ and XiO have similar approaches. For Pinnacle$^3$, the layer spacing is variable and dependent on the Bragg peak width. The system places layers such that the distal 80% of the shallower layer matches the proximal 80% of the next deepest layer (in later versions the user can alter the distal percentage value to alter the spacing, but this was not possible in the version we tested). In XiO, the spacing is also variable and equal to the Bragg peak width (defined in commissioning, with a definition of the user's choosing) times by a peak width multiplier (an integer value selected by the user during planning). To ensure consistency with Pinnacle[3], we set the Bragg peak widths in commissioning as the 80%–80% width of each pristine Bragg peak, and used a peak width multiplier of 1. For Eclipse, the layer spacing cannot be defined in the same way, but it can be configured with the following options during commissioning: fixed distance (in mm) available throughout the energy range; fixed change in energy (in MeV); fixed distance (in mm), determined based on the range sigma of the highest or lowest energy per field; or variable distance, equal to the range sigma of the next highest energy layer multiplied by a user-defined multiplication factor. In our study, the variable distance option was selected in an attempt to maintain consistency with Pinnacle[3] and XiO. The range sigma used in Eclipse only accounts for the energy spread of the initial beam, whereas the Bragg peak width used in Pinnacle[3] and XiO also accounts for the range straggling. To determine what multiplication factor should be used, we experimented with beams of different range and modulation, both with and without a range shifter. The further the layers are spaced apart, the larger are the dose ripples along the beam direction. The closer they are together, the more layers there are (hence longer delivery time) and the greater becomes the sensitivity to the minimum spot MU problem (more layers mean fewer MUs per layer and, hence, per spot), which leads to spikes and troughs as spots below the minimum MU threshold are either rounded up to the minimum MU or rounded down to zero. The dominating phenomenon depends on the range and modulation. However, because Eclipse's beam configuration only allows for the choice of a global value, it was found that using four times the range sigma was a good compromise between these competing factors across all the beams studied.

For Eclipse and XiO, the available spot positions are defined by a 3D rectangular grid passing through the isocenter, within the target boundaries (and margins). Pinnacle[3] defines 2D square grids for each layer, starting from the left-hand side of the target (in the beam's eye view), such that the available positions of successive layers are often offset. An attempt was made to minimize any variation in planners' ability with each system by asking for feedback on the plan quality from planners working for the individual vendors.

#### F. Optimization options and dose calculation parameters

Optimization involves minimizing some variable that quantifies target volume coverage and OAR sparing. During this optimization process, the stopping tolerance was set to a suitably small value relevant to each system (0.001 in Eclipse, $10^{-5}$ in Pinnacle[3], and 0.0001% in XiO), so that an optimal dose distribution was ensured, but also that the optimization did not take longer than 30 minutes (the maximum number of iterations was never reached). Although an important factor in optimization processes, differences between computing powers of the individual workstations made timing comparisons infeasible.

Each system has different optimizers and/or options available, as summarized in Table 3. In this study, the plans were quantified through dose volume histogram (DVH) metrics, so for systems in which there is a choice, we selected the optimizer that gave the best dosimetric plan.

Table 3. Optimizers available in each TPS.

| *TPS* | *Optimizer* | *Comments* | *Used in Study* |
|---|---|---|---|
| Eclipse | Simultaneous spot optimization (SSO) | Better DVH (IMPT or SFUD) | Yes |
| Eclipse | Conjugate gradient (CG) | Smoother distributions (IMPT or SFUD) | No |
| Pinnacle[3] | IMPT | IMPT or SFUD plans | Yes |
| XiO | Beamwise optimization of fluence | SFUD plans only | Yes |
| XiO | Full intensity-modulated proton therapy | IMPT plans only | No |
| XiO | Sequel beamwise optimization | Compromise between SFUD robustness and IMPT coverage | No |

No robustness options and/or features were tested. Eclipse has two available optimizers: (i) the simultaneous spot optimization (SSO) algorithm, which is based on a scanning optimization algorithm;[(43)] and (ii) the conjugate gradient (CG) algorithm. Both optimizers can produce SFUD and IMPT plans, but only SSO was used in this study as it gives better DVHs.[(44)] The Pinnacle$^3$ optimizer, IMPT, allows for the production of either SFUD or IMPT plans (only the former was tested in this study). A robustness option and the creation of erroneous patient setup scenarios is also possible, but was not tested in this study. XiO has three options when optimizing: (i) beamwise optimization of fluence, which produces SFUD plans; (ii) full intensity-modulated proton therapy, which produces IMPT plans; and (iii) sequel beamwise optimization, which provides a compromise between the robustness of SFUD plans and the coverage of IMPT plans. In this study, only the first option was utilized as only SFUD plans were produced. A smoothing option was available during the optimization, but was not employed.

The dose was calculated on all systems with a grid size of 2.5 mm, using each system's most accurate dose calculation algorithm (for Eclipse this is 'Proton Convolution Superposition', for Pinnacle$^3$ 'Proton PBS', for XiO 'Pencil Beam Algorithm'). All three systems account for heterogeneities and model nuclear interactions. For full details of the different algorithms, the reader should consult the relevant literature for Eclipse,[(44)] Pinnacle$^3$,[(45)] and XiO.[(25)] As stated in the introduction, the accuracy of each dose calculation algorithm was not assessed due to the scale and depth required by the task.

The monitor units (MUs) of each system were normalized to be identical for a uniformly irradiated $5 \times 5 \times 5$ cm$^3$ cube, 5 cm deep within a water phantom. The MUs needed to be comparable between systems because the uniformity of spot weights was to be assessed (see Material & Methods section G below). The 5 cm depth was chosen to ensure that the available 74 mm (water-equivalent) thick range shifter was included, as the targets in all patients extend more proximally than the lowest available energy and thus require the use of this device.

### G. Evaluation

To avoid discrepancies in the final volume building of DVHs, datasets were exported with consistent dose bin widths (0.1 Gy) and analyzed independently using CERR, the Computational Environment for Radiotherapy Research.[(46)] For each patient, a set of parameters was computed from the DVHs.

The uniformity and distribution of spot weights from each field was also analyzed using 3D spot maps. The spot MUs were determined by multiplying the spot weight by the calibrated MUs for the given field. A parameter, C, was defined to allow quantitative comparisons between systems:

$$C = \left(\frac{\Sigma[w(i) \times d(i)]}{\Sigma w(i)}\right) \quad (1)$$

where $w$ is the weight and $d$ the distance from the isocenter for a spot $i$. The distance is calculated using the x and y coordinates and energy (converted to a water-equivalent Bragg peak depth) for each spot.

## III. RESULTS

Table 2 details the mean target coverage and OAR sparing for the three systems for a variety of parameters typically assessed during treatment planning. A mean value for each statistic is given in units of Gy, with an error defined by the standard error on the distribution across the ten patients. Although the errors are larger for the OARs because each case requires different organs to be spared, the values are still useful for comparison between systems (each system had the same range of cases). In brackets are the numbers of patients (out of ten) that fail to meet the higher/lower constraints. One-way analyses of variances (ANOVAs) were calculated between the three systems for each metric, with p-values shown in the far right column. Figures 1 and 2 provide a graphical representation of these metrics, with the boxplots showing the distribution of results across the ten patients. The edges of each box are formed by the 75th ($q_3$) and 25th ($q_1$) percentiles; the whiskers extend to the most extreme value that is not an outlier; points are considered outliers if their results are greater than $q_3 + w(q_3 - q_1)$ or smaller than $q_1 - w(q_3 - q_1)$ (where w is set to 1.5) and are shown by crosses; and the median is shown by the black circle within the box. As an illustration, Fig. 3 shows dose distributions for Patient 8 planned on all three systems.

To assess the uniformity and distribution of spot weights of the plans produced by each system, the weight of each spot (normalized to each system's mean) was plotted against its absolute distance from the isocenter. This is shown in Fig. 4(a), together with the calculated values for C (Eq. (1)), for all patients and all fields. The mean weight of all spots, at every 1 mm, is shown in Fig. 4(b) for all systems.

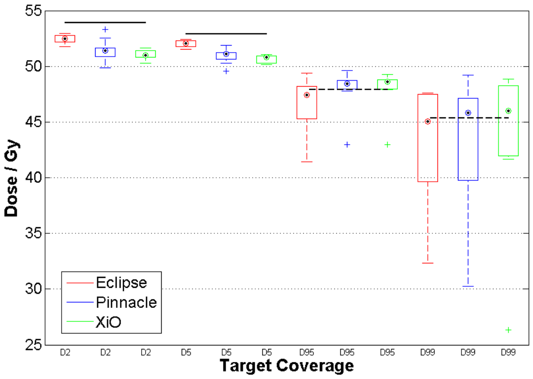

Fig. 1. Target coverage statistics for the ten patients, for each system. Boxplots are as described in the text, with the constraints of each statistic (from Table 2) shown by the black lines (solid for upper constraints, dashed for lower constraints).

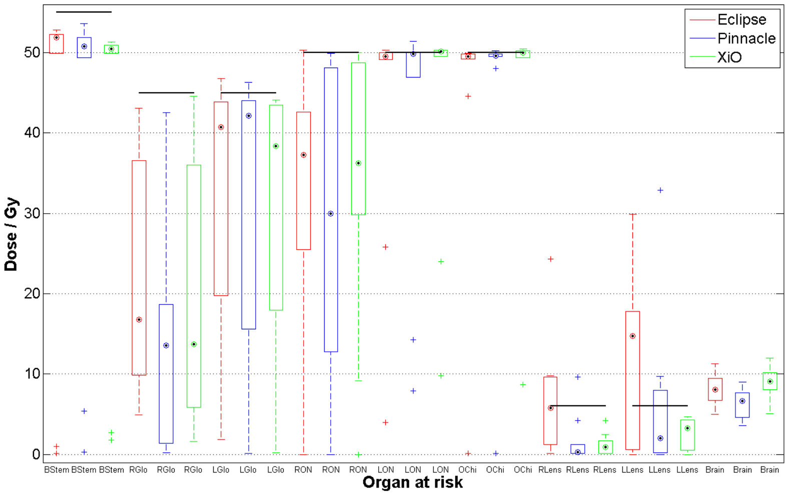

Fig. 2. OAR doses for the ten patients, for each system. Maximum doses are shown for the PRVs of (left to right) brainstem, right globe, left globe, right optic nerve, left optic nerve, optic chiasm, right lens, and left lens. Mean doses are shown for the healthy brain. Boxplots are as described in the text, with the upper constraints of each OAR (from Table 2) shown by the corresponding solid black line.

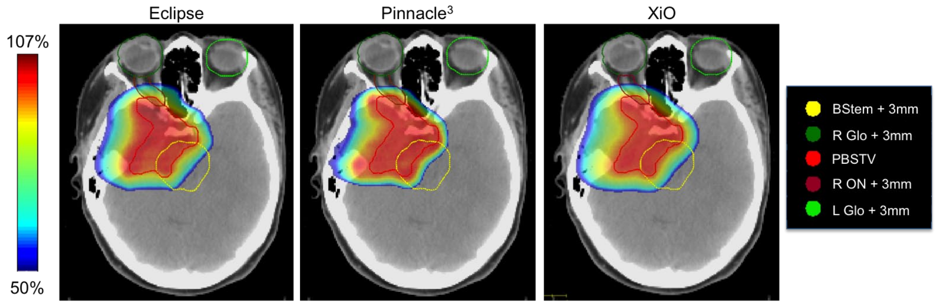

FIG. 3. Dose distributions for Patient 8. Structure abbreviations can be found in Table 2.

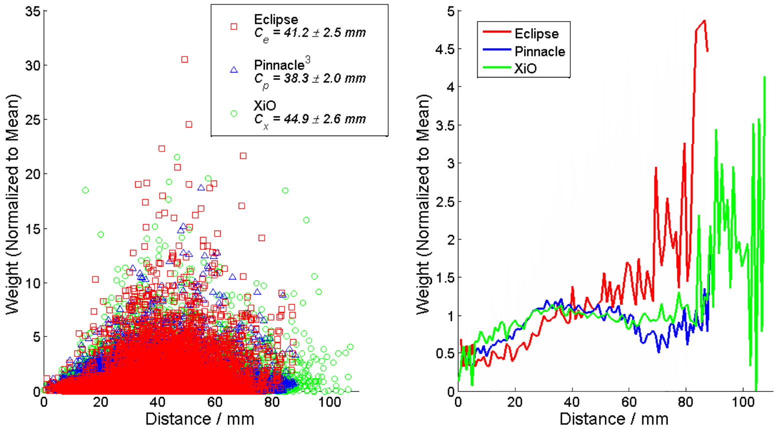

FIG. 4. (Left) Relationship between the normalized spot MUs and distance from the isocenter for all patients, for Eclipse (red squares), Pinnacle³ (blue triangles), and XiO (green circles). The calculated values for C (Eq. (1)) are displayed on the figure for Eclipse ($C_e$), Pinnacle³ ($C_p$), and XiO ($C_x$). (Right) The mean weight of all spots, at every 1 mm, for each system (same color scheme).

### IV. DISCUSSION

#### A. Overview

The aim of the study was to compare the dose distributions produced by three proton TPSs — Eclipse, Pinnacle³ and XiO — with a common set of planning guidelines, specifically for meningioma patients. The use of spot scanning protons for meningiomas has been shown to be beneficial,[37] and the cases selected challenged each system as there were often many overlapping structures. Plan differences could be attributed to system differences, which would both inform new proton centers deciding which TPS to purchase and encourage further development of proton TPSs.

With a consistent planning strategy, all systems showed a good capacity to produce satisfactory plans that sufficiently respected the constraints for both the target and OARs, despite difficult and conflicting objectives, with large overlapping regions. The operation of these different systems and the options available to the planner do differ, but the final results were similar.

Statistically significant differences were found for the high target doses, D2 ($p = 1.7 \times 10^{-4}$) and D5 ($p = 5.6 \times 10^{-7}$), the maximum dose for one of the lenses ($p = 0.024$) and the mean brain dose ($p = 0.022$). Although not significant, there was a general tendency for Pinnacle³ to deliver lower OAR doses (as can be seen in Fig. 2). Mean integral doses outside the PBSTV, across all patients, were found to be 4.4 ± 1.5 Gy in Pinnacle³ (mean ± standard deviation), compared to 6.0 ± 2.2 Gy in Eclipse and 6.3 ± 2.3 Gy in XiO. This can also be seen in Fig. 3, with Pinnacle$^3$'s dose distribution showing marginally better conformality to the tumor than those of Eclipse and XiO. A possible reason for this is the flexibility of available spot positions, which are more staggered than the fixed 3D grids available in Eclipse and XiO, as illustrated in Fig. 5. Other possible reasons, such as different energy layer spacings and other system differences, are detailed below.

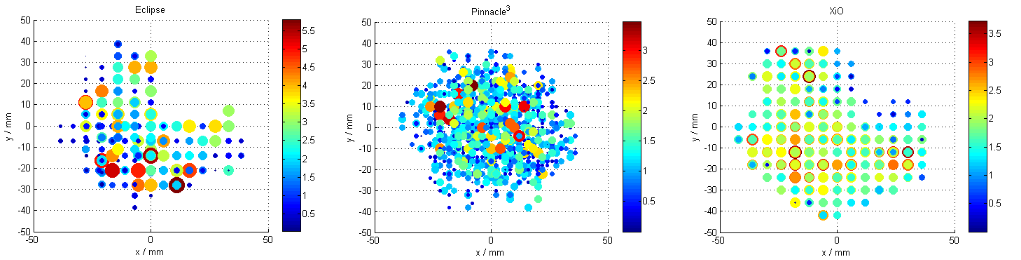

Fig. 5. Beam's eye views of the spot distributions, with the spots in all layers overlaid for (left) Eclipse, (middle) Pinnacle$^3$, and (right) XiO, for a particular patient and field. Each spot map has been normalized to its own mean, with spot color and size proportional to the spot weight. The color scale range differs between the three plots.

### B. Energy layers

As stated in the Materials & Methods section above, the layer spacing could not be defined in a consistent manner for all systems. Attempts were made to make the resultant layer spacing of each system consistent, however this proved difficult. It was found (by analyzing mean ± standard error across all patients) that Pinnacle$^3$ (21 ± 2) used fewer energy layers than XiO (31 ± 2). A variable spacing of four times the range sigma was used in Eclipse, but this was perhaps not high enough, as the average number of layers (17 ± 1) was lower than both Pinnacle$^3$ and XiO. As mentioned in the Materials & Methods section, this value had to be selected in the commissioning (prior to the planning) and it led to a potential source of disadvantage to Eclipse. In the plans produced, the number of spots per layer was similar: Eclipse (68 ± 9), Pinnacle$^3$ (86 ± 12) and XiO (79 ± 11) (mean ± standard error across all patients).

### C. System differences

Eclipse and Pinnacle$^3$ allow multiple fields to be simultaneously optimized as separate SFUD fields, whereas XiO requires separate optimization of each field, with half the prescription dose and half the tolerance doses to the OARs, followed by summing of the different field doses at the end. This effectively doubles the optimization computational time.

In each system, the same objectives are applied to all fields, but they can be scaled by setting the relative beam weights. Pinnacle$^3$, however, is the only system that has an option to allow the relative weighting of the fields to be adjusted by the optimizer (i.e., following optimization, the relative field weightings are adjusted from those initially set by the user). Sometimes one field can better spare an OAR while delivering more dose to the target than another, but it may not be clear to the planner the precise relative weighting of the beams that should be used to maximize this. This is a useful option and could be a potential explanation for the generally lower OAR doses discussed in Discussion section A.

It is known that, for this version of Eclipse, the SSO optimizer has a tendency to form one or two highly weighted spots. Ordinarily, the planner would assess the effect of removing these, but no postprocessing of spot weights was completed in this study in order to specifically test the algorithms. This occurred less in Pinnacle$^3$ and XiO.

### D. Spot uniformity and distribution

An assessment was made of uniformity and distribution of spot weights between TPSs. A parameter, C, was defined to quantify this difference, which involves analyzing the spot weight as a function of the distance from the isocenter (Eq. (1)). In Fig. 4(b) it can be seen that, although not significant, Pinnacle$^3$ has a more uniform distribution of spot weights than Eclipse and XiO. This is backed up by the parameter C in Fig. 4(a), which has a lower value for Pinnacle$^3$ (38.3 ± 2.0 mm) than for both Eclipse (41.2 ± 2.5 mm) and XiO (44.9 ± 2.6 mm). Although not verified in this paper, it is hypothesized by the authors that such a metric could be used as a heuristic measure of field robustness. For a field to be robust, the highly weighted spots should generally be located further from the edge of the target (and thus closer to the isocenter), so that changes in patient position and range uncertainty within a given field are then less critical to the target coverage and dose to the surrounding OARs. This would lead to a lower value of C. Verification of this hypothesis, however, and the determination of a threshold value for C, are beyond the scope of this work.

It should be added that this measure of robustness is only really applicable to SFUD plans and is in contrast with the desire for an optimal plan. The larger degrees of freedom in IMPT plans allow for generally better coverage and OAR sparing,[19] but it has also been shown that IMPT techniques, such as distal edge tracking (in which the intention is to deliver highly weighted spots to the distal edge of the target), are less robust to uncertainties.[38,47] A variety of methods to handle such uncertainties have been suggested,[48–53] including the reduction of the intensity of spots close to tissue heterogeneities,[54] which is along similar lines to our hypothesis.

### E. Study limitations and future work

As stated in the introduction, comparing different TPSs is a very difficult task due to the many interconnected components. Any study trying to perform such a task will have limitations and, as such, care should be taken to rank systems based on these preliminary results.

In an ideal study, the dose calculation would be performed in a single or independent engine. This is difficult to achieve in practice, however, as dose calculation is a necessary part of the optimization process and the two cannot be easily disentangled. As mentioned in the methods, the dose calculation algorithm differs between systems and it is inevitable this will impact on the plans. For instance, it may be possible to attribute the higher OAR doses in Eclipse and XiO to deficiencies in their dose calculation algorithms leading to an overestimation of the lateral penumbra, as has been reported to be the case for uniform scanning proton therapy.[26] A thorough, detailed assessment of the dose calculation algorithms of each system is thus necessary to validate our findings.

As stated in the Materials & Methods section, time is an important factor in the optimization process, but this could not be assessed due to the hardware differences between systems. Also, the layer spacing could not be controlled in a consistent way for each system, and the resulting number of layers available to each optimizer differed.

To calculate the distance for the quantitative metric C (Eq. (1)), it was necessary to convert the spot energy to a water-equivalent range, which does not necessarily correspond to the physical coordinate of the pristine peak in the patient relative to the isocenter. It should also be noted that each system has different options available during optimization, as stated in the Materials & Methods section, which may improve the result of C, (conjugate gradient optimization (Eclipse), robustness option (Pinnacle$^3$), smoothing function (XiO)); however, none of these was tested.

The plans were made robust to range uncertainties using a uniform 5 mm expansion from the CTV; however, a full assessment of robustness requires shifts in the patient position and systematic and statistical variations in the patient density and chemical composition. Such assessments could not be carried out within all TPSs tested, and it is a procedure that we would like to carefully control in an independent scheme (such as in MATLAB). This is an area of future work.

The study only looked at meningioma brain treatments using fixed horizontal SFUD fields. It is anticipated there would be bigger differences in the performance of each system when producing full-gantry IMPT plans, and this is suggested as an area of future work. How systems cope with different treatment sites will be of interest because of the different OARs and heterogeneity issues that must be considered. Also, as with any TPS comparison study, comparison results age quickly because of the continual evolution of each system.

### V. CONCLUSIONS

The study compared the plans produced by three proton TPSs — Eclipse, Pinnacle$^3$, and XiO — for the treatment of meningiomas with an SFUD horizontal fixed beam arrangement. Few statistically significant differences were found, but Pinnacle$^3$ generally gave lower OAR doses, with an integral dose outside the target 27% lower than Eclipse and 30% lower than XiO, on average, across all patients. Possible reasons for this are the flexibility of available spot positions and the option that the optimizer can adjust the relative weighting of the two fields; however, the dose calculation algorithms of each system must be assessed in future works to validate our findings. Pinnacle$^3$ was found to distribute its spots more uniformly than Eclipse and XiO. In highlighting the differences between the systems we believe the study will prove to be useful both to new proton centers and to the improvement of the TPSs themselves.

### ACKNOWLEDGMENTS

We would like to acknowledge the support and guidance of Maura Kirk at the Hospital of the University of Pennsylvania, who assisted with beam geometry selection and other planning enquiries. The authors would also like to thank the patience, support, and plan verification provided by Varian, Philips, and Elekta. This project was funded equally by the Engineering and Physical Sciences Research Council (UK) and Ion Beam Applications (Louvain-La-Neuve, Belgium).

## Supporting information

Supplementary Material
