# Supplementary material for: A comparison of the dose distributions from three proton treatment planning systems in the planning of meningioma patients with single‐field uniform dose pencil beam scanning
Source: J Appl Clin Med Phys. 2015 Jan 8;16(1):86–99. doi: 10.1120/jacmp.v16i1.4996 (PMC5689989; doi:10.1120/jacmp.v16i1.4996)
Supplement: Supplementary file 1 — Supplementary Material [file ACM2-16-086-s001.doc]

**Performance analysis of the optimization algorithms of three proton treatment planning systems in the planning of meningioma patients with single-field uniform dose pencil beam scanning**

**AUTHORS**

**Paul J Doolan^1*^, Jailan Alshaikhi^1,2^, Ivan Rosenberg^2^, Chris G Ainsley^3^, Adam P Gibson^1^, Derek D’Souza^2^, El Hassane Bentefour^4^ and Gary J Royle^1^**

*^1^Department of Medical Physics and Bioengineering, University College London, London, U.K.*

*^2^Department of Radiotherapy, University College London Hospital, London, U.K.*

*^3^Department of Radiation Oncology, University of Pennsylvania, Philadelphia, PA, U.S.A.*

*^4^Ion Beam Applications (IBA), 3 Chemin du Cyclotron, Louvain la Neuve, B-1348, Belgium*

*Corresponding author: [paul.doolan.09@ucl.ac.uk](mailto:paul.doolan.09@ucl.ac.uk)

**RUNNING TITLE**

Performance analysis of the optimization algorithms of three proton treatment planning systems

**ABSTRACT**

With the number of new proton centres increasing rapidly, there is a need for an assessment of the available proton treatment planning systems (TPSs). This study assesses the performance of the optimization algorithms in complex meningioma patients of three proton TPSs: Eclipse, Pinnacle^3^ and XiO. Proton treatment plans for ten patients were produced on each system with a pencil beam scanning, single field uniform dose approach, using a fixed horizontal beamline. All 30 plans were subjected to identical dose constraints, both for the target coverage and organ at risk (OAR) sparing, with a consistent order of priority. Beam geometry, lateral field margins and lateral spot resolutions were made consistent across all systems. Few statistically significant differences were found between the target coverage and OAR sparing of each system, with all optimizers managing to produce good plans despite strict constraints and overlapping structures. Pinnacle^3^ did, however, give generally lower OAR doses and also demonstrated the most uniform distribution of spot weights.

**KEY WORDS**

Proton therapy, particle therapy, treatment planning
